# Beyond Intention: An Exploration on How Precision Fermentation Technology for Animal-Free Cheese may Affect the Meaning of Cheese

**DOI:** 10.1007/s41055-025-00197-7

**Published:** 2026-01-27

**Authors:** Renske Bouma, Kasper Hettinga, Mariana Hase Ueta, Zoë Robaey

**Affiliations:** 1 Food Quality and Design Group, Wageningen University & Research, Wageningen, the Netherlands; 2 Philosophy Group, Wageningen University & Research, Wageningen, the Netherlands

**Keywords:** Cheese, Precision fermentation, Novel food, Relational ontology, Mediation theory, Ethics by design

## Abstract

How we think about food results from a myriad of aspects, ranging from production to consumption. The emergence of new food technologies brings new modes of production that could inaugurate new meanings to foods. This paper discusses precision fermentation technology, as it could enable us to make cheese with the same nutritional and sensorial properties as traditional cheese, without animals. This brings promises of lowering impacts of diets, while still consuming a highly nutritious food, cherished by many. Food design often focuses on nutritional and sensorial aspects of the product, but the meaning of food is dependent on more than its physical properties. As a means for normative reflection and guidance for the design of novel food, this paper offers a framework to systematically reflect on possible effects of technology on the meaning of food in our lives, and applied to animal-free cheese. How might the broad meaning of cheese change when it is produced by precision fermentation? What implicit intentions are involved, and what effects might happen beyond intention? The relational ontology framework for food by Deane Curtin is combined with the anticipating mediation framework for technology by Peter-Paul Verbeek. The combined framework is used to imagine several possible effects, building on the positionality of a food scientist developing this technology and with a background in Dutch dairy production. These anticipated effects are by no means exhaustive but form a starting point for continued imagination, reflection and discussion, especially for those involved in the development of novel foods.

## Introduction

Precision fermentation of milk protein could enable us to make animal-free cheese with the same nutritional and sensorial properties as traditional cheese, without the cow (Hettinga and Bijl 2022). This could allow people to lower the impact of their diet, while still consuming a highly nutritious food that is cherished by many. Even though the environmental and animal-welfare impact of dairy has been under attention and milk consumption has gone down, cheese consumption retains a steady upwards trend (Dagevos et al. 2023; Wolf et al. 2020). The appeal of cheese is illustrated by the fact that despite their concerns with animal-based food, vegetarians eat on average not less, but almost twice as much cheese as meat-eaters (Bradbury et al. 2017), particularly due to its taste and versatility (Docherty and Jasper 2023). Correspondingly, 41% of people trying a vegan diet report missing cheese the most (Veganuary 2021). The design of high-quality animal-free cheese could therefore be a game-changer in the transition to a more sustainable food system.

Food science often focusses on nutritional and sensorial aspects of the product (see e.g. (Birch and Bonwick 2018; Bouma et al. 2025; Grahl et al. 2018)), but the meaning of food is dependent on more than the physical object. To create a starting point for normative reflection and guidance for the design of animal-free cheese, this paper systematically reflects on the possible effects of the technology on the meaning of cheese in our lives. The reflection takes the positionality of a food scientist developing this technology, in combination with a background in Dutch dairy production. How might the broad meaning of cheese change when it is produced by precision fermentation? What implicit intentions are involved, and what effects might happen beyond intention?

Precision fermentation may affect the meaning of cheese, as it allows the animal-free production of a unique type of milk protein, called casein. This protein is responsible for the high protein and mineral content of traditional cheese, as well as its typical taste and texture (Fox et al. 2017). Plant-based cheese alternatives developed so far are incapable of matching traditional cheese, as plant proteins are not able to mimic the milk protein functionalities (McClements and Grossmann 2021). Hettinga and Bijl (2022) described how precision fermentation can lead to the production of nature-identical milk proteins. The genetic code of the milk proteins has been uncovered through biological studies of cows and other mammals, and with this, synthetic DNA sequences can be built. With gene-editing technologies, the synthetic DNA sequence can be incorporated into the genome of microbes such as yeasts or bacteria. During fermentation the microbes will then produce milk proteins that are in the basis molecularly identical to animal milk proteins. The proteins are separated from the microbes, and further processed into dairy products such as cheese. In this way, precision fermentation allows for the usage of the unique milk proteins, without involving cows or other mammals, leading to the concept of animal-free cheese (Hettinga and Bijl 2022).

In this article the product and the production technology are discussed together, as these are necessarily and strongly intertwined: no product exists without production. When we talk about either cheese or technology, it encompasses both the production method (precision fermentation) and the product itself (animal-free cheese). In Sect. 2, we first set the stage and present current philosophical explorations at the intersection of philosophy of food and philosophy of technology, as well as reflect on our methodology. In Sect. 3, we start our exploration of the meaning of cheese by broadening the current meaning of cheese beyond the physical through a relational ontology and with help of the three categories defined by Curtin (1992): literal, social and symbolical. In Sect. 4, we use the same framework to make the intended changes in the meaning of cheese explicit, from the perspective of a food scientist working with precision fermentation. However, to imagine the possible effects of the new technology beyond intention, an elaborated framework is needed. In Sect. 5 we draw on Curtin’s categories in combination with the anticipating mediation framework of Verbeek (2011). The combined framework is used in Sect. 5.1–5.3 to imagine several possible effects of the new technology in a wide scope, ranging from platypus cheese to farmer-cow collaborations to cheese-based polarization. Section 6 concludes this article and looks forward to a future where the broad meaning of food and the multitude of possible effects of new technologies are taken into account while designing novel foods, to ensure that good intentions do indeed lead to desirable outcomes.

## How to Engage With Novel foods?

### Philosophical Explorations at the Intersection of Food and Technology

In this section, we present current philosophical explorations at the intersection of philosophy of food and philosophy of technology. This will allow us to situate the contribution of this paper, that builds on mediation theory and relational ontology of food to understand the possible effects of novel foods. This suggests that food no longer is simply the result of technological processes, but is -in fact- technology. In this paper, we take the view that the process and the outcomes of technology cannot be separated for the case of precision fermentation cheese and similar novel foods. This is supported by the lack of philosophical frameworks to engage with the effects of novel foods.

The intersection of philosophy of food and philosophy of technology has traditionally been studied through technological developments in agricultural practices, such as the use of Genetically Modified Organisms (GMOs) for seeds, and other chemical and mechanical developments that became part of the Green Revolution. These developments invite questions on the limits of our interventions, discussions on technological optimism and technofixes, and more generally how technology in agriculture challenges a set of existing practices around food and health and their related institutions (see (Scott 2018; Thompson 2009)). These prominent accounts do not address the advent of precision fermentation as a genetic engineering method for producing food ingredients that are then assembled into food products, like casein in animal-free cheese.

Scholarship outside of philosophy has taken interest in animal-free cheese, in particular relating to consumers (Broad et al. 2022) and policy (Mendly-Zambo et al. 2021). In ethics, the moral desirability of animal-free cheese has been discussed (Milburn 2018) and is echoed in the debates on imitation meat as (Bacchini and Bossini 2023). Most headway on novel foods such as animal-free cheese has been made at the intersection of philosophy of food and philosophy of technology through ontological arguments. For instance, ontological categories call for public deliberation in establishing novelty of novel foods (Piras 2024). An alternative approach suggests bringing an artefactual view of food concepts like meat, or cheese, to take functions into account in the definition of novel foods, and therefore make their novelty less disruptive (Bacchini and Bossini 2025).

While these accounts are important, they are not necessarily action-guiding when it comes to supporting food technologists in their design choices, which brings us to consider mediation theory (Verbeek 2011) as a constructive framework to reflect on the design of novel foods. This post phenomenological framework has not yet been used to account for food^1^, and this paper innovates by complementing it with a relational view on food by Curtin (1992). This leads to the construction of a framework for reflection and anticipation, made by and for food technologists that we present in detail in Sect. 5. We thereby suggest a way to think of alternatives on how novel foods are currently being developed. This adds to the literature on disruptive technologies and helps questioning technofixes (Sætra and Selinger 2024). The framework invites to reflect on speculative futures with different configurations on ownership of food, labor of farmers and animals, and our relationship to nature. This relates to different visions of synthetic biology (Tarnowski and Pansera 2024).

Before presenting the framework, we add some methodological notes on making use of the first author’s positionality, in the context of fostering reflection in innovation processes.

### Situating the Reflection with the Designers

Understanding that this emerging technology of precision fermentation comes from a context of situated knowledge (Haraway 1988) helps us reflect on our positionalities as food designers involved in the technological production processes, while taking into consideration the first author’s background in cheese making in the Netherlands, where cheese making is valued and celebrated as part of the culture and national identity. According to Haraway (1988), all knowledge is situated in its context and, in this way, shaped by its social and cultural experiences and based on personal experiences and intersectional positionality. By bringing positionality into consideration, we recognize that all actors, such as cheese makers, are embedded in intersectional dynamics. In other words, the overlapping and interacting social categories (such as gender, race, class, sexual orientation, family background, etc.) matter for knowledge production. This approach challenges the notion of universal and objective truths by considering its epistemic context and opens up the reflexive opportunity to learn from the actors involved in the social-technical processes. The present discussion is inspired by the long-standing Personal Narrative scholarship in anthropological tradition (Walley and Elliott 2025). The personal narrative of an actor engaged in designing novel foods provides a snapshot of the context of food production transformation. In the context of emerging technologies and transformation in food systems, bringing the perspectives of the actors who are impacting in the food design and will be impacted by its application in society is central to reflecting on the new meanings of this food. By inscribing personal experiences, we aim to contribute to a non-alienated knowledge production that provides a generative space to discuss technology-led transformations in food systems, and the changing meanings of food.

In this paper, the first author developed the analytical framework, and used it to reflect and analyze personal experiences, or situated knowledge, in cheese making and novel food design. This was further substantiated by existing literature on these issues. The work was supported by discussions with the other authors, who contributed by situating the contribution within the field of food ethics, making sense of the positionality of the analysis, widening the scope of ethical consideration, and corroborating the interpretations. There were no points of contention between the authors.

## A Multifaceted Meaning of Cheese

Before diving into the meaning of cheese, it is useful to take a step back and have a look at how foods acquire their meaning. For this we use a relational ontology approach; meaning is a result of the relationship between food and us. A food is shaped through the combination of the edibility of the material and the willingness of a person to consume it. Deane Curtin described in his essay *Food/Body/Person* that a participatory relation can be understood as a defining relation (Curtin 1992); both food and person are (partly) defined through their relationship. Curtin defines three categories of participatory relations: literal, social/political and symbolical/spiritual. These categories are used below to describe a current meaning of cheese, drawing from the first author’s personal experience as cheese maker, scientist and consumer and as a Dutch granddaughter to dairy farmers. A multifaceted meaning of cheese is summarized in Table 1, and described in the next paragraphs.

**Table 1:** A current meaning of cheese using the three categories defined by Curtin (1992) through reflection from the first author’s positionality

	Literal	Social	Symbolical
Current meaning	Nutritious Delicious	Large ecological impact International trade Business model of growth; intensification of animal rearing	Dutch national identity

### A Literal Meaning of Cheese

The literal meaning of food relates to the physical product, and the effect it has on the body (Curtin 1992). Cheese is rich in protein, fat and minerals, supplying the body with high quality nutritional components as well as energy (Geurts 2022; O’Brien and O’Connor 2004). In the act of eating the cheese, the person senses such aspects as the smoothness of a soft cheese or the rich flavor of a ripened cheese. Literally, cheese is nutritious and delicious.

### A Social Meaning of Cheese

The social/political meaning of food relates to the effects of our eating practice on the workings of our society in a broad sense, including environmental impact, markets and finances (Curtin 1992). The production of cheese has a large environmental footprint, in use of water and energy as well as in emissions of greenhouse gasses, contributing to global warming (Canellada et al. 2018; Kim et al. 2013). Dutch semi-hard cheese production is associated with 8.5 kg CO2 equivalent per kg cheese, of which 65% originates from the milk production (van Middelaar et al. 2011). This high impact is due to the concentration of nutrients -for 1 kg of cheese 10 L of milk is needed- and the use of cattle. The greenhouse gas emissions from Dutch dairy farming originate from the animals digestive system (44%) and manure (28%), as well as from feed production (20%) (FAO 2017). Next to the global climate, the local nature is directly affected by extensive land use and emission of nitrogen and phosphorus, risking reduced water quality and loss of biodiversity (Van Calker et al. 2005).

Market-wise it is important to realize that the large-scale production of cheese consists of an international chain. The Netherlands export yearly more than a billion kg of cheese (ZuivelNl 2024). Feed is imported to the Netherlands from all over the world (Wang et al. 2018), affecting the economies and land use choices of these countries too; international trade has contributed to global welfare and alleviated poverty (Anderson 2014), but the demand for animal feed crops like soy have also led to large-scale deforestation (Paim 2021). Lesser known examples of chain parties are the manure traders, bull breeders, calf auctioneers and slaughterhouses, providing livelihood to all involved.

All chain parties have to deal with their respective financing structures. Investors tend to expect and demand growth. In the Netherlands, the bank Rabobank is known as the farmers bank, financing 85% of the agro-industry and thus yielding much power on food production methods through their decision framework with which they determine which investments are sound and which are not (van Bekkem et al. 2018). In the past decades, many investments have been put towards intensification, increasing the number of cows per farm from an average of 25 in 1975 to 110 in 2023 (Genootschap ter Bevordering van Melkkunde 2019; ZuivelNl 2024). Now that the Dutch government realizes that the amount of cattle has to decrease to respect ecological boundaries, the bank is experiencing social backlash for their promotion of growth (Nieuwenhuis and Keultjes, 2023; NOS, 2019; van Bekkem et al. 2018). Socially, cheese in the Dutch context currently means a large ecological impact, international trade and an outdated business model of growth.

### A Symbolical Meaning of Cheese

The symbolical and spiritual meaning of food relates to identity and community (Curtin 1992). The food we eat expresses our values and what group we belong to (Backer et al. 2019). To eat cheese, particularly on bread for lunch or in cube-form at parties, is often described as typically Dutch. In a recent Dutch survey, 93% of the respondents said that cheese is part of the Dutch national identity (GfK Panel 2024). The symbolic relationship between the Dutch and dairy does not limit itself to consumption, but also includes production; the same survey showed that 89% of the respondents thinks that dairy farms fit well in the Netherlands (GfK Panel 2024). The cow-filled meadows are considered a typical Dutch sight, which is no surprise considering that more than 80% of the 1.6 million Dutch dairy cows graze outdoor (ZuivelNl 2024). In short, both consumption and production of dairy -and cheese specifically- symbolize the Dutch national identity.

To conclude, the current meaning of cheese can be seen as multifaceted, with literal, social and symbolical dimensions, as exemplified in Table 1. The described current meaning of cheese forms the starting point from which we will discuss changes in meaning upon introduction of the new technology.

## The Intended Change

A new technology is developed with an intention in mind. We use Curtin’s framework to describe the intended change of precision fermentation on the multifaceted meaning of cheese from the perspective of the designer. For this technology, the designer’s perspective is that of the first author, a food scientist working on animal-free cheese. The exact intentions may well differ between different designers; to the best of our knowledge no research has been published on this topic. The intentions, as summarized in Table 2 and described below, should therefore be considered as relevant, but not unanimous or exhaustive.

**Table 2:** Current and intended meaning of cheese in the three categories as described in the relational ontology framework by Curtin (1992) through reflection from the first author’s positionality

	Literal	Social	Symbolical
Current meaning	Nutritious Delicious	Large ecological impact International trade Business model of growth; intensification of animal rearing	Dutch national identity
Intended meaning	No change	Reduced ecological impact No animal-use	De-normalize animal-use Techno-solutionism

The designer explicitly intends no change in the literal meaning of cheese. As precision fermentation produces proteins that are in the basis molecularly identical to animal protein, it should be possible to produce cheese that is just as nutritious and delicious as traditional cheese. The body would and should not be able to notice the difference between traditional and animal-free cheese.

Socially and symbolically, the food scientist’s intentions are implicit but present. Socially, the food scientist intends to lower the ecological impact of cheese production. By not using cows, especially methane emissions and land use are expected to drastically decrease, lowering global warming potential and increasing local nature opportunities. Not using cows is also a goal in itself, as cows are sentient beings that are not able to live their best lives in the current intensive farming practice. This goal also has a symbolic dimension, as animal-free cheese is intended to bring attention to and de-normalize the role of animals in our food system. New technologies can change our moral intuitions and inform techno-moral change (Nickel et al. 2022). Besides this, the intent for the technology is to be a symbol for techno-solutionism, the optimistic idea that research and technology can help us towards a better future (Sætra and Selinger 2024).

To summarize, Curtin’s framework allows us to reflect on and render explicit the current and intended multifaceted meaning of cheese. However, technology often has more effects than those intended by the designer. In order to design the new technology responsibly, one needs to be aware of the possible effects of the technologies beyond our intentions.

## Imagining the Effects of Technology Beyond Intention

To imagine the possible effects of the new technology, we will elaborate the food-related framework of Curtin with one geared towards new technologies. For this we use Verbeek’s framework for anticipating mediations of technology (Verbeek 2011). Technology can mediate both our actions (pragmatic) as well as our interpretation of the world (hermeneutic). Verbeek describes three forms of mediation: designer delegation, technology emergence and user appropriation. The overview of the combined framework is schematically represented in Table 3, and explained in more detail below.

**Table 3:** Overview of the combined framework of Curtin (1992) and Peter-Paul Verbeek (2011)

	Literal	Social	Symbolical
Current meaning (Sect. 3)	Starting point of the food scientist	Broadening to a multifaceted meaning of food	
Intended meaning/Designer delegation (Sect. 4)	Explicit intentions of the food scientist	Implicit intentions made explicit	
Technology emergence	Imagining *pragmatic* mediation effects of the new technology user = consumer (Sect. 5.1)	Imagining *pragmatic* mediation effects of the new technology user = chain parties (Sect. 5.2)	Imagining *hermeneutic* mediation effects of the new technology user = consumer Sect. (5.3)
User appropriation			

The designer delegated mediation as described by Verbeek can be seen as the intended effects from the food scientist described in the previous paragraph. The designer, i.e. food scientist, explicitly intends to delegate to the precision fermentation technology to affect the literal meaning of cheese in a specific manner (same nutrition and taste). However, implicitly the designer also intends to delegate to the technology social and symbolic meaning. These implicit intentions were made explicit in Sect. 4, made possibly by the broadening of the meaning of food from the literal towards a multifaceted meaning using the categories of Curtin (literal, social, symbolical) in Sect. 3.

In the next sections, we will describe possible effects beyond intention by moving from the context of design to the context of use. To do so, one needs to anticipate how the technology is implemented in practice (emergence), and how people use the technology (appropriation). We will try to anticipate these two forms of mediation for the three different categories of Curtin. For both the literal and social meaning of cheese, in Sect. 5.1 and 5.2, we will focus on pragmatic mediations of the new technology. These categories relate mostly to what actions we can and will do, and it is of interest to anticipate how the new technology will change our options. Finally, for the symbolical meaning of cheese, in Sect. 5.3, we will consider hermeneutic mediation, as symbolism is highly related to how we interpret the world.

Using the combined framework, one can start to anticipate several changes of the meaning of cheese when the animal-free cheese would be introduced. The intention is not to feign future telling abilities; it is entirely possible that unexpected effects emerge or that multiple seemingly contradictory effects will be seen in parallel. The imagined effects, described below and summarized in Table 4, serve to exemplify the anticipations that can be made using this framework and to start imagination, reflection and discussion.

**Table 4:** Excerpt of the described current, intended and anticipated meaning of cheese through reflection from the first author’s positionality. The list is exemplary and by no means exhaustive of all possible meanings of cheese

	Literal	Social	Symbolical
Current meaning	Nutritious Delicious	Large ecological impact International trade Business model of growth; intensification of animal rearing	Dutch national identity
Intended meaning/Designer delegation	No change	Reduced ecological impact No animal-use	De-normalize animal-use Techno-solutionism
Technology emergence	Different products *e.g. platypus cheese*	International trade Accessible nutrition	Dutch innovation Consumer citizenship
User appropriation	Rebound effect; more consumption	Empowerment of farmers Different role for animals *e.g. farmer-cow collaboration*	Cheese-based polarization Techno-fix

### Anticipating the Literal Meaning of Cheese

#### Technology Emergence; Scientific Limitations and Market Priorities

In the literal sense, the technology might emerge differently than intended, despite this being the explicit focus of the designer. The intention is to produce a cheese with the same nutritional and sensorial properties as traditional cheese. However, research and development is ongoing on this topic, and it is unknown whether this goal is 100% attainable (Lucey 2022). The proteins produced by precision fermentation are not yet shaped as they are in milk, so to mimic them completely extra process steps will be needed and even then some limitations might remain. Shaping the protein is subject of current research by multiple scientists (Antuma et al. 2025; Che et al. 2025; Fan et al. 2024; Wang et al. 2024). Besides protein, also fat and micronutrients affect the properties of cheese (Fox et al. 2017). Even though proteins are the main structuring agent in cheese and particularly important for nutrition, differences in the other ingredients of cheese could mean that animal-free cheese will turn out somewhat different from the traditional cheese it is trying to mimic. However, one could argue that even if a complex specialty like Grana Padano or Burrata cannot be mimicked, it would still be possible to successfully mimic cheese in a general sense, due to the existent large diversity in cheeses and their accepted variety in sensorial and nutritional properties.

Besides scientific limitations, market priorities are likely to influence technology emergence as well. Precision fermentation might be relatively expensive, particularly at the start, driving producers to incorporate less protein in their products consequently lowering the similarity with traditional cheese. Producers might not even consider similarity desirable, as creating new products could be used to emphasize the novelty. More fundamentally, precision fermentation could be used to produce different proteins altogether. With this technology it would be possible to create whole new types of protein, that have not existed before, to create new food products. Legal approval of novel food is however a complicated business, including the need to supply toxicological, allergenicity and nutritional information (Fytsilis et al. 2024). For whole new proteins the amount of research needed would be especially large, as their effect on the human body would be entirely unknown. It is therefore more likely that precision fermentation will be used to produce and possibly optimize proteins that already exist in nature. Still, this means that one could produce milk proteins from any one mammal, e.g. orangutan, dolphin or platypus. These proteins have somewhat different functionalities than the milk protein from cows, so a cheese made with platypus milk protein is likely to taste and digest differently than traditional cheese. It cannot yet be said whether such a novel cheese would have specific benefits, but it might be produced for the market simply for the sake of novelty and to showcase our abilities.

#### User Appropriation; Subjective Experience and Rebound Effects

Besides the way that the technology is implemented in practice, how the final user appropriates the technology is also of importance. The designer expects no change in sensory experience or nutrition, but this might prove false. One can imagine that if people are wary of the technology, this might lead them to experience the cheese differently, as information about a product can affect how we perceive it (McClure et al. 2004; Mukherjee and Sahay 2018). Nutrition-wise, the assumption of the designer is that people will eat the same amount of cheese as they have done before. Consumption can however increase either by people eating more cheese than before, or by (re)introducing cheese in their diet. Reintroduction is expected to be relatively small, as a recent consumer study in the UK showed that only a minor portion of the animal-free market share (1.2% of 17.7%) is expected to come from those that did not buy cheese before (Slade and Zollman Thomas 2023). Rather, high cheese consumption and flexitarianism have strong correlations with the willingness to buy animal-free cheese (Slade and Zollman Thomas 2023; Zollman Thomas and Bryant 2021). It can be imagined that these people, who like to eat cheese but are conscious about their food choices, will take the opportunity to eat more cheese when they find that it has been freed from its negative moral connotations - a techno-moral scenario similar to that imagined by Swierstra et al. (2009) for the obesity pill. Cheese is an energy dense food, so higher consumption quickly leads to significant higher energy intake. Besides a different nutrition than anticipated, this rebound effect through user appropriation would affect the social meaning of cheese; a higher consumption leads to a higher footprint, while the intended aim was to decrease it. Besides this, more social effects can be imagined.

### Anticipating the Social Meaning of Cheese

#### Technology Emergence; Relevance of Production Chain and Efficiency

The type of food production chain that emerges upon introduction of the technology will influence the social meaning of cheese. In spite of the molecular similarity to the animal product, the production process of the novel cheese is completely different, does not involve the cows, and does not necessarily have to be conducted in rural areas. In this sense, this new production method demands different inputs and biomaterials which inaugurates new supply chains. The type of chain will depend on the crop required for fermentation, but it is not yet known which that will be. Ideally a range of different crops would lend itself for fermentation, to increase flexibility over seasons and locations. It is envisaged that current dairy farmers could switch from keeping cows to growing the required crops, resulting in economically viable farmers and locally produced crops. However, it might not be possible for the farmers to grow the required crops on their lands due to the local soil and climate. Even if it would be possible, the government might be more interested to use the land for housing, nature or industry, especially in a highly populated country as the Netherlands. Growth of crops and final production may become spatially decoupled, as is now largely the case for animal feed production (Wang et al. 2018). The current international trade of crops could consequently be transformed. Important feed crops such as soybean and maize might not be usable for fermentation, and the producing countries -mainly USA, Argentina and Brazil (Wang et al. 2018)- could experience decline in export if they do not change production. Concurrently, countries that do produce the crops preferred for precision fermentation could see a rise in export, which could have both positive, e.g. economic welfare (Anderson 2014), as negative effects, such as inequalities of food access, pollution and loss of biodiversity (Howard 2022). Besides the displacement of production, it is also important to consider the labor transitions that these technologies could entail. According to Morais-da-Silva et al. (2022) these technologies could impact the labor market not only by creating new jobs, but also by demanding different expertises. With precision fermentation of milk protein, there would be less need for dairy farmers. Farmers might however appropriate the technology to their benefit, an option that is discussed further in Sect. 5.2.2. 

The intention is to diminish the ecological impact of cheese production, but this will depend on how the process will emerge (Hettinga and Bijl 2022). Besides the supply of crop, the ecological impact of precision fermentation depends on location of production, processing choices, protein yield and process efficiency (Behm et al. 2022). Efficiency of production does not only affect the ecological impact, but also determines at which scale it is possible to run the fermentation, with additional social effects. If it is possible to run the fermentation efficiently in small scale with little extra processing steps, small fermentation plants could be built around the globe to supply the local community. In this way it might be possible to produce dairy at places where before cows did not prosper, such as cold mountain ranges like North-West China or in urban areas around the globe (van Biezen 2024). This could allow more people, notably children, to access this nutritious food, contributing to distributional and cosmopolitan justice (Moritz et al. 2024). Local production could lower the price through elimination of expensive (cooled) transport. Price has been shown as an important indicator for child dairy consumption (Headey 2023). The author found that with higher buying power, child dairy consumption increases more rapidly than that of eggs and meat, probably because dairy is often seen as particularly nutritious for young children (Headey 2023). Whether accessible nutrition will indeed be part of the new meaning of cheese, will greatly depend on the scalability and economics of the emerging technology, as well as on the choices of those who take charge of the production chain.

#### User Appropriation; Choices Along the Chain

To imagine the social implications through user appropriation, the first question to answer is who the user of the technology is in this context. As said before, the technology encompasses both the product and the production method. For the literal implications, i.e. the meaning for the body, it made sense to focus on the consumer as the user, in relation to the product. When one wants to discuss the social and political implications, i.e. the meaning for society, it makes sense to focus on the food production chain, with producers as users, in relation to the production method. The users of the technology can then be considered those parties that are in charge of the precision fermentation activities, but who that will be is yet unclear.

Multiple companies are working on developing the technology, such as Formo, PerfectDay and Remilk. When they are successful, they can choose to keep the information silent, share their knowledge freely or patent it. Patenting goods is something that should not be taken as a given due to the moral implications of patenting (Lever and Timmermann 2025). In the case of the first author’s research project, the company Those Vegan Cowboys is involved and a combination of information strategies is used; the company is likely to file patents, but underlying and related knowledge found by the university researchers is shared in open access research articles. The idea -but not necessarily the reality- of patenting is that it increases innovation as investors know that if they are successful, they can earn their investment back by a temporary monopoly (Hall and Harhoff 2012). The patent-holder can use the information themselves, but providing licenses and services to others is likely to lead to more impact and profit, as well as improve technological justice, a term introduced by Moritz et al. (2024). The costs and conditions of such licenses will determine who will be able to take charge of the new technology.

The social effects will depend on the choice for a centralized, decentralized or distributed production system (Soice & Johnston, 2021), as well as who it is that makes this choice. Farmers might take the opportunity to install a fermentation tank on their premises and feed the microbes with locally produced crops, similarly as envisioned for cultivated meat (RESPECTfarms 2024). They could choose to use the proteins themselves on-site, and produce animal-free farmer’s cheese, or alternatively transport milk proteins to a local co-owned cheese factory. The dairy industry in the Netherlands historically has a cooperative structure, where farmers together own the factories. Such a cooperative could also choose to invest in a centralized precision fermentation factory. This would allow for scale benefits of increased efficiency, both economically and environmentally. Dairy cooperatives exist in a range of sizes, from 10 up to 1000’s of farmers, which understandingly affects the level of influence of an individual farmer on the factory’s management. Alternatively, venture capitalists could invest in precision fermentation factories. Farmers could then still be involved, by selling the required input for the fermentation, but they would not be in control. The technology could thus be used by farmers to reinvent their business, empowering them, while it could also be used by investors to gain profit, with farmers taking a backseat. For the Dutch context, Hase Ueta, Robaey, and Kunze (2025) investigated how dairy farmers perceive their future with (or without) these technologies in the transformations of their own farms, and propose to actively include dairy farmers in the transformation of cheese production.

The choices of farmers will not only influence their own role in the food production chain, but also that of the animals. From the Dutch perspective, the reduction in cattle is topical because of the surplus of manure produced; there is too little land for too much fertilizer, correlating with a high livestock density (MacDonald et al. 2011). In other countries, however, this imbalance is not as extreme or even reversed; in eastern Europe for example there is a deficit (MacDonald et al. 2011) meaning cow’s manure is a gift rather than a burden. Not only geographically, but also in different farming practices the role of animals is different. In mixed farms, animals are kept together with a mix of different crops; manure can be used locally and immediately, and the animals behaviour sustains certain ecosystems. Specifically, grasslands grazed by cows form a biological niche for insects and meadow birds, including the Dutch national bird the godwit (Hooijmeijer et al. 2021). Eliminating cows entirely would greatly affect our food system as well as local nature. It can therefore be imagined that farmers that produce crops for precision fermentation will not completely eliminate cows, but instead give them a different role, acknowledging them as a form of animal justice (Moritz et al. 2024). One can imagine that cows will remain with their calves in small herds that fit their natural behaviour, cooperating with the farmer in the sense that one supplies the land to live on and the other the fertilizer (manure) to their mutual benefit: fertile land abundant in both food and feed. This can be taken into consideration under the ethical lens of animals as laborers (see Blattner et al. (2019)), and what happens in terms of recognition once that labor is displaced. Precision fermentation cheese could then mean, instead of animal-free, animal-liberated cheese; a new social as well as a symbolic meaning of both cows and cheese.

### Anticipating the Symbolical Meaning of Cheese

#### Technology Emergence; Symbol for Dutch Innovation and Power of Consumption

Depending on how the technology emerges, cheese as a symbol for the Dutch national identity could be strengthened. If the technology is developed successfully by a Dutch company, such as WildWestLand, a collaboration between Those Vegan Cowboys and Westland Cheese, the technology could become a symbol for the great abilities of Dutch innovation. This image fits with the Dutch position within the EU as ‘Innovation leader’ (European Commission 2023).

The technology is developed on the inherent assumption that problems can be solved through steering consumption behaviour, and as such symbolizes our role as ‘consumer citizens’: the power and responsibility to steer society by our consumption choices (Kallhoff 2013). This attitude risks however to diminish other aspects of civilian life, such as the role of political engagement (Hermsen 2019). It may also hide the responsibility of other actors like the government and industry, through placing all responsibility on individuals, known as ‘responsibilisation’ (Littler 2011). The extent of these effects will not only depend on the technology, but also greatly on the ‘consumer citizens’ themselves, i.e. the user appropriation.

#### User Appropriation; Symbol for Moral, Sentimentalism and Lazy Habits

Vegan consumers could use the animal-free cheese as symbol to distinctly oppose traditional cheese, emphasizing their negative moral judgement on the dairy industry. This could lead to resistance from non-vegans. People tend to react negatively towards morally motivated persons, defending against anticipated moral judgement, an effect known as do-gooder derogation (Minson and Monin 2011). This effect would come on top of the existent ridiculed portrayal of vegans, posed as overly sentimental and ascetic (Cole and Morgan 2011). Who wants to be part of this group? Each dietary choice contributes to our social identity; food is a group marker, symbolizing to what community we belong (Backer et al. 2019). The choice between traditional and animal-free cheese could therefore become symbolically loaded and polarized, preventing some from making the switch to the new cheese.

Consumers that do feel positively towards the new cheese can use it to lower their impact without changing their habits. The new cheese could thus be considered a techno-fix: a technological way to apparently solve a social problem without the need to change behaviour (Sætra and Selinger 2024). Techno-fixes are distinct from techno-solutionism, as the latter is a progressive optimistic approach focusing on disruptive changes made possible by technology, while techno-fixes are conservative in nature (Sætra and Selinger 2024). Consumers could appropriate the technology as symbol for the lack of need to change behavior; techno-fix attitudes were associated with a reduction of behavioral intentions, which was not the case for techno-optimistic attitudes (Cologna et al. 2024). Contradictorily, a technology meant to improve our environmental impact might thus impede the behavioral change also required to reach said goal.

## Conclusion

This article started with the question: how might the broad meaning of cheese change when it is produced by precision fermentation? We have imagined a multitude of changes in the literal, social and symbolical dimensions of the meaning of cheese when precision fermentation is introduced, through intended designer delegation, technology emergence and user appropriation. The presented possible changes illustrate that novel food technology can have effects beyond those intended, and they are by no means exhausted. All readers are welcomed to imagine more possible changes to the meaning of cheese, from their situated knowledge, for example by the choices of agents not yet discussed in this article such as governments, supermarkets and agricultural lobby groups. Additionally, we invite the reader to use the presented method, combining mediation theory and relational ontology of food, to explore and anticipate intended and unintended effects of other novel food technologies. Having frameworks dedicated to these types of food innovations is important for anticipating the possible changes, forming normative reflection and doing ethics by design. This makes it possible to steer novel food technologies already during development, to prevent unintended negative effects and to advance the intended positive effect: a sustainable food system with respect for nature, producer and consumer.

## Data Availability

No datasets were generated or analysed during the current study.

## Footnotes

1 It is important to note that much work has been done in phenomenology and food, albeit not novel foods.
